# Stress responses and experiences of surgical trainees in simulation-based training of advanced laparoscopic procedures in highly realistic environments

**DOI:** 10.1186/s41077-025-00400-z

**Published:** 2026-01-09

**Authors:** Maria Suong Tjønnås, Sébastien Muller, Johannes Tjønnås, Mariann Sandsund, Solveig Osborg Ose, Cecilie Våpenstad, Gjermund Johnsen

**Affiliations:** 1 Department of Neuromedicine and Movement Science (INB), Faculty of Medicine and Health Sciences, NTNU, Norwegian University of Science and Technology, Trondheim, N-7491 Norway; 2 Department of Health Research, SINTEF, SINTEF Digital, P.O. Box 4760, Torgarden, Trondheim, NO-7465 Norway; 3 SINTEF Digital, Mathematics and Cybernetics, SINTEF, P.O. Box 4760, Torgarden, Trondheim, NO-7465 Norway; 4 National Research Center for Minimally Invasive and Image-guided Diagnostics and Therapy (MiDT), St. Olavs Hospitalt, Trondheim University Hospital, P.O. Box 3250, Prinsesse Kristinas gate 5, Torgarden, Trondheim, NO-7006 Norway

**Keywords:** Physiological stress response, Medical simulation, Procedural training, Heart rate variability, Advanced laparoscopic skills, Surgical training course, Saliva cortisol, Subjective stress ratings, STAI-6, Semi-structured interviews

## Abstract

**Background:**

Simulation-based training facilitates learning of advanced laparoscopic surgical procedures. Such procedures are challenging to master due to their technical complexity, which can elicit stress responses in surgical trainees. Previous research has demonstrated the impact of stress on trainees’ learning processes in skills lab. However, there is limited data comparing trainees’ stress responses during advanced procedural training using box-trainers in skills lab, to those experienced during operating training in realistic simulation environments. This study aims to explore the physiological responses, self-reported stress responses, and experiences of stress of surgical trainees during advanced laparoscopic procedural training in two different simulation environments. Insights into stress mechanisms may suggest improvements for the design of future training courses.

**Methods:**

This observational study explored participants’ stress experiences through semi-structured interviews and investigated their stress responses by measuring the heart rate variability, saliva cortisol levels, and trainees’ self-reported stress using a validated instrument. Participants performed advanced laparoscopic procedures on box-trainers and live animal models in operating room settings.

**Results:**

Twelve experienced surgical trainees were included in the study. No differences were observed for physiological parameters between training activity on the box-trainer simulator and live animal models. In interviews, trainees reported experiencing higher stress levels during procedural training in operating room environment. The main themes related to elevated stress were realism and functional task alignment, perceived level of risk, and interpersonal dynamics in simulation environments. The trainees perceived the increased stress response levels as beneficial for their focus and for performing advanced procedures.

**Conclusion:**

In this study, no significant differences were identified in trainees’ physiological or self-reported stress responses across the two simulation settings. However, qualitative interview data revealed that trainees perceived greater stress when training with animal models in highly realistic operating room environments, suggesting the potential educational value of such immersive simulation environments.

**Supplementary Information:**

The online version contains supplementary material available at 10.1186/s41077-025-00400-z.

## Introduction

Surgical techniques used in laparoscopic procedures require specific sets of psycho-motoric skills [1]. These techniques are not innate, and surgical trainees need to invest a substantial amount of training hours in order to master these skills [2]. The use of highly realistic simulation environments may be effective in the training of complex surgical procedures [3]. Advanced laparoscopic procedures can be practiced using box-trainer simulators. These simulators allow training on both animal and artificial tissue, while real laparoscopic instruments provide natural haptic feedback during practice [4]. With the laparoscopic tower setup, trainees can work with video camera images, creating a visually realistic environment that closely mirrors actual surgical conditions [5]. In addition to box-trainer systems, training may be conducted in highly realistic environments, such as procedures performed on live animal models within full-scale operating room settings. These environments closely replicate real patient operations, offering conditions that prepare trainees for advanced procedures [6]. Both box-trainer and high-fidelity simulations are typically supervised by expert laparoscopic surgeons, ensuring that trainees receive guidance while performing complex procedures.

Performing laparoscopic surgery is associated with high stress levels, particularly among surgical trainees [7, 8], and this need to be considered in training design. Previous research has described how trainees experience stress during training courses due to several factors related to simulation training tasks. These factors include issues with task realism, lack of tactile feedback, time pressure, and self-imposed pressures, all of which can induce stress responses during laparoscopy training courses [9].

Stress response is a complex phenomenon involving both physiological and psychological mechanisms [10, 11]. In cognitive appraisal theories, when an individual encounters a threat or challenge, they will automatically appraise the situation [12, 13]. First, they assess the specific demands required to overcome the threat or challenge and then look at the resources available to meet those demands. If the resources are enough, the situation is viewed as a manageable challenge, and the stress is seen as eustress [14]. On the other hand, if the resources are insufficient, the situation is viewed as a threat, and the stress is seen as distress [12, 13]. When a situation is appraised to be a threat or a challenge, immediate changes in the nervous, cardiovascular, endocrine, and immune systems are observed [15]. These changes are the neurobiological stress responses of the body, characterised by the activation of the sympathetic nervous system (SNS), which can increase heart and respiration rates within seconds [15]. The SNS and the hypothalamic-pituitary-adrenocortical (HPA) axis produce stress hormones [16]. The SNS stimulates the adrenal medulla, which in turn produces catecholamines, while the HPA axis stimulates the secretion of the hormone cortisol. This increase in cortisol levels has a direct influence on various neurological and musculoskeletal functions in the human body [17]. Previous research has demonstrated how heightened stress response levels may have both detrimental and beneficial impacts on surgeons’ learning, performance outcomes, and patient outcomes [8, 18, 19].

Data on stress responses during advanced procedural training using box-trainer simulators compared to live animal models in realistic operating room settings remain limited. Moreover, stress in simulation-based laparoscopic training courses is underexplored, despite its potential relevance. A greater understanding of the stress responses levels in simulation environments could inform training strategies for surgical trainees, educators, and clinical staff.

This study aimed to investigate stress responses in simulation-based training by assessing perceived, physiological, endocrine, and self-reported stress responses among surgical trainees during advanced laparoscopic procedures in two distinct simulation settings.

## Methods

This prospective, observational study, was conducted at a national research centre for minimally invasive surgery in Norway. As a part of their specialist training, surgical trainees undergo mandatory training in advanced laparoscopic techniques and procedures. This training typically occurs between the third and fifth year of a standard five-year training program. This advanced training is organised as seminar-based courses which last for three consecutive days.

### Recruitment of participants

The study was open to all surgical trainees who had previously taken and passed the mandatory course in basic laparoscopic techniques and were enrolled in the advanced laparoscopic procedure course. The exclusion criteria included medical conditions that affected or influenced the heart rate variability or hormone levels, i.e., arrhythmia or ongoing pregnancy. Trainees were invited to participate in the study by oral invitation at the beginning of each course. Written consent was obtained from all participants prior to joining the study. Participants characteristics, work experience, laparoscopic practice, and simulation training experiences were collected through a questionnaire (Additional file 1). One surgeon instructor was interviewed and gave contextual information about these courses and shared the perspectives of the instructors. Data were collected at five courses from December 2019 through February 2024.

### Simulation sessions

The simulation sessions were divided into three parts. Simulation sessions in course day 1 contained introductory tasks, while simulation sessions on course day 2 and course day 3 contained training on box-trainers or operating on live animal models. On course day 1, all participants took part in an introductory simulation training session. This session focused on becoming familiar with laparoscopic techniques, equipment, tasks, and procedures for the following days of the course. One session consisted of training specific laparoscopic techniques and procedural tasks on pig organs. This session was conducted in the skills laboratory. The other session involved training in specific laparoscopic procedures on live, anaesthetised pig in a full-scale operating room setting. The operating room facilities, a part of the university hospital research centre, were certified for medical research involving animals and adhered to the ethical guidelines for the use of animals in research [20]. The latter two simulation sessions lasted four hours and were conducted on course days 2 and 3.

#### Simulation of advanced laparoscopic procedures using a box-trainer and a laparoscopic tower

The simulation task consisted of performing advanced laparoscopic procedures (resections and anastomosis) on pig stomach and small intestines. The trainees were partnered up for this task, one acting as the main operating surgeon, while the other would act as the assistant surgeon. Trainees would take turns serving as the main and assistant surgeons, with each session as the main operating surgeon lasting approximately 60 min. Each team used a setup that included a box-trainer and a laparoscopic tower, which contained surgical equipment typically used in the operating room, in addition to a side table stacked with surgical needles, tread and gloves. Surgeon instructors were present during the sessions to observe and guide the trainees.

#### Simulation-training of advanced laparoscopic procedures on live animal models in full-scale operation room settings

The simulation task consisted of performing advanced laparoscopic procedures (resections and anastomosis) on pig stomach and small intestine in a highly realistic operating room environment. The trainees were divided into teams of three or four persons, where one acted as the main surgeon, another as the main assistant surgeon, and the remaining as assistant surgeons providing technical support for the main surgeon. Each turn as main surgeon lasted for approximately 60 min. A surgeon instructor guided the trainees through all the surgical procedures. Trainees would rotate and take turns as the main operating surgeon.

A detailed description of the simulator type, scenario design, orientation to simulators and the environment, simulation procedures, and feedback/debriefing is provided in Additional file 2, the supplementary Table S1 in accordance with Cheng et al. (2016). *Key Elements to Report for Simulation-Based Research* [21]. The simulation sessions lasted for four hours.

**Fig. 1:**
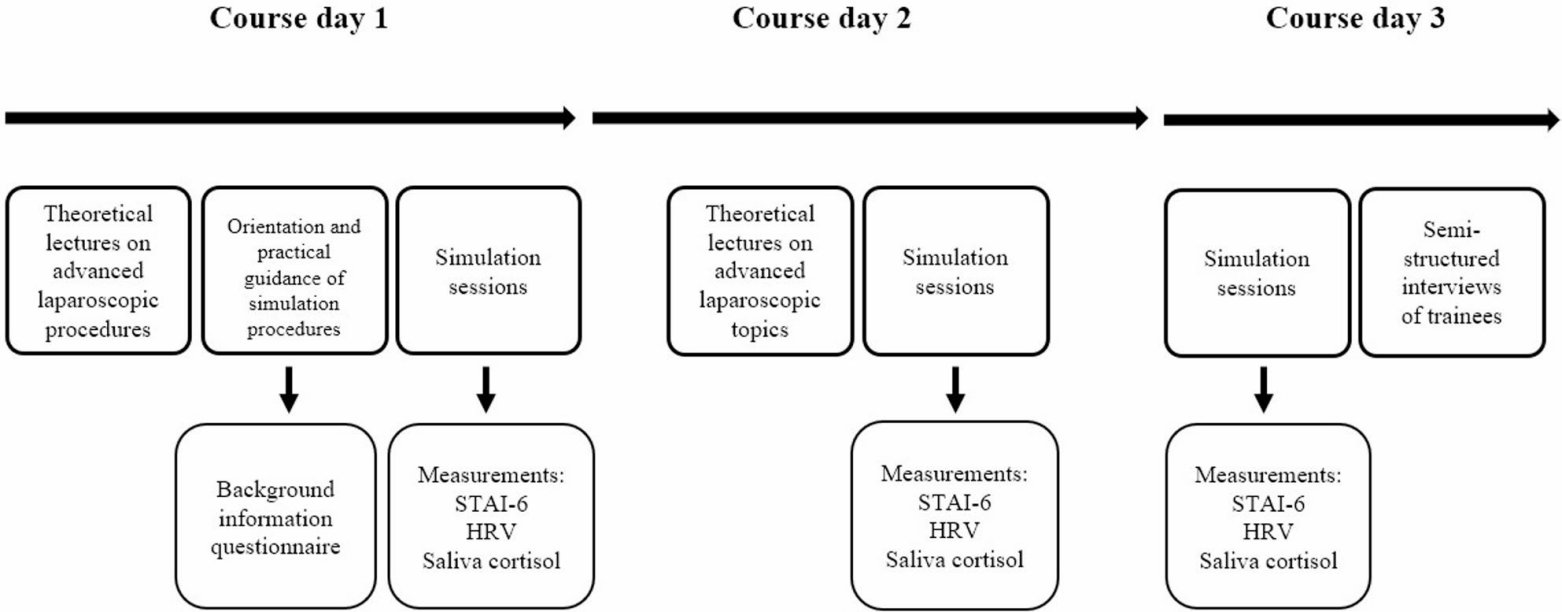
Schematic overview of study design, showing the simulation-based training course timeline, simulation sessions, stress measurements and interviews. HRV = heart rate variability, and STAI-6 score = State-Trait Anxiety Inventory scores, the six item version by Bekker et al. 1992 [24]

The study was reported according to the STROBE Statement reporting guidelines with extensions for simulation-based research in Additional file 3 [21]. The interviews were recorded and transcribed verbatim. The transcripts were organised for analysis in Excel using the method by Ose [22]. The findings from the interviews were reported and detailed in accordance with the COREQ guidelines-checklist for reporting qualitative research in Additional file 4, the Table S2 [23]. Fig. 1 shows a schematic overview of the study design with the training course timeline (upper part), indicating interventions of this study, including stress measurements and interviews.

### Data collection

#### The physiologic stress response measures

This study used HRV metrics and one endocrine marker to assess physiological stress responses in surgical environments [25].

The HRV analysis were based on R-R intervals from ECG signals, representing the time between successive R-waves [26]. The R-R intervals served as the foundational data for the analysis of HRV data and the calculations of the HRV metrics presented.

The root mean square of successive differences of R-R intervals (RMSSD) reflects vagal tone and is highly correlated with high-frequency HRV. High RMSSD values indicate a strong respiratory sinus arrhythmia component, in addition to reflect high parasympathetic cardiac activation [27].

Standard deviation of the R-R intervals (SDNN) reflects all the cyclic components that contribute to variability during the recording period. The SDNN measures the autonomic influence on HRV. High SDNN values indicate a high parasympathetic cardiac activation [28].

The activity of the SNS primarily influences the low-frequency (LF) band of HRV, which ranges from 0.04 to 0.15 Hz. In contrast, the high-frequency (HF) band, spanning 0.15 to 0.4 Hz, predominantly reflects parasympathetic nervous system activity [27, 29]. Elevated LF activity combined with reduced HF activity is typically associated with high stress levels, whereas lower LF and higher HF activity suggest a state of low stress [30].

Saliva cortisol served as an endocrine marker for stress [31]. The concentration of cortisol in saliva is similar to that in serum, with a delay of 2–3 minutes [32].

#### Subjective measures of stress

To measure self-reported stress, this study used the State-Trait Anxiety Inventory- six item (STAI-6), a short version of the STAI questionnaire (Additional file 5), which consists of six statements [24]. Participants indicated their level of agreement with each statement using a four-point Likert scale. The statements reflect six distinct anxiety states, which can be associated with cognitive, emotional, and physical reactions to stress [33].

### Collection

Stress response measures (HRV metrics, STAI-6 scores, and saliva cortisol) were recorded before, during, and after training on the box-trainer and the operating room simulation sessions. ECG signals were recorded using an ECG recorder (The Actiwave Cardio, CamNtech, Cambridge, UK). The recorder was attached to the participant’s chest upon arrival at the training facilities. ECG signals were recorded while participants rested and during simulation sessions. In resting conditions, participants sat on a chair without talking for 20 to 45 min, followed by standing for 20 to 30 min. The participant’s ECGs were recorded continuously throughout all simulation sessions. Participants were video recorded using action cameras (GoPro HERO7, GoPro, Inc., San Mateo, US) to exclude artifacts and non-simulation activities and annotate simulation activities. Saliva cortisol was collected using a Salivette swab (Salivette Cortisol. Sarstedt, Numbrect, Germany). Participants received written and verbal instructions on restrictions and allowances. Participants were instructed to avoid certain foods, naturopathic preparations, and all nicotine products for 24 h before sampling. They were also asked to abstain from eating, drinking coffee, tea, or soft drinks for 60 min prior, and to refrain from strenuous physical activity during the course. Saliva samples were collected at rest and during simulations, specifically when participants acted as lead surgeon performing the main procedure. Samples were collected between 1 PM and 3 PM, with a maximum time variation of one hour per participant. Samples were collected 20–25 min into rest and simulation sessions to avoid carry-over effects [31], stored at 5 °C, centrifuged, and frozen at −70 °C until analysis. The STAI-6 was completed during rest and key simulation tasks, including knot tying, anastomosis, and vessel repair. Semi-structured interviews, guided by a study-specific protocol (Additional file 6), were conducted individually on day 3 after both simulation sessions, lasting 20–40 min.

### Questionnaires and semi-structured interviews

For this study, a combination of subjective data from the questionnaires and the interviews were analysed. A qualitative content analysis approach was chosen as this can be applied for both qualitative and quantitative data, and the method aims to draw information from data to understand its context, to provide knowledge, new insights, or a clear view of facts [34–36]. An inductive analysis was conducted involving coding, category creation, and abstraction [35].

### Data analysis

The ECG recordings were post-processed using MATLAB^®^ (MATLAB^®^, The MathWorks, Inc. Natick, US) and Python™ (Python™, Python Software Foundation, Fredericksburg, US) software. The ECG sampling rate was set at 256 Hz for the recordings. Ectopic beats, artifacts and data errors were removed from the ECG datasets using an HRV-analysis package [37]. The video recordings of the simulation activities were manually annotated for each participant and their simulation activities. The last 30 min of the simulation activity segment, during which participants acted as the main surgeon, were used for HRV analysis.

A laboratory centre at a university hospital analysed the saliva samples. Analysis procedure of saliva cortisol included liquid extraction and analysis with high pressure liquid chromatography (Agilent 1290 high-pressure liquid chromatograph with Agilent 6465 Triple Quad LC/MS-MS detector).

The total STAI-6 scores were calculated by reversing the positive items (calm, relaxed, content) and summarized in accordance with to analysis method by Marteau and Bekker [24].

### Statistical analysis

Normality was assessed using the Shapiro-Wilk test and Q-Q plots. Data were reported as mean differences and standard deviations (SD) for continuous variables, and as counts and percentages for categorical variables. HRV data were presented as means ± SD, with significance set at *P* < 0.05. Repeated measures ANOVA compared HRV data and cortisol measures across resting conditions, simulation activities, and environments, with Bonferroni correction for multiple comparisons. Significant effects were further examined using Tukey’s post hoc test and pairwise *t*-tests. STAI-6 scores were compared using the Mann-Whitney U test and pairwise *t*-tests. An alpha level of 0.05 was used throughout. All analyses were performed using IBM SPSS Statistics 29 for Windows.

### Qualitative analysis

The questionnaire text and interview data were read and categorised [38]. The categories were then grouped under broader headings by merging similar categories into broader categories [35]. Categories were created to describe the stress response experiences of trainees in the context of advanced laparoscopic procedural training. All relevant data was interpreted and compared, and after iterative discussions among researchers, the data was placed in subcategories [34]. The outcomes of the analysis were themes with subcategories describing the stress responses related to training with different simulation modalities and settings. Detailed description of the qualitative analysis is provided in Table S2 (Additional file 4).

## Results

Of the 46 surgical trainees that were invited to join the study, twelve trainees voluntarily participated, eleven men and one woman. The mean age was 33 years (± 3.7 years), with an age range of 28 to 40 years. Participants had an average of 4 years of experience (± 2.0 years), with a range from 2 to 5 years as surgical trainees. Participants characteristics, work experience, and experiences with laparoscopic techniques, procedures, and simulations are presented in Table 1.

**Table 1:** Participant characteristics, work experience as surgical trainees, previous experience with laparoscopic procedures and techniques, simulations, computer games and fine motoric activities (*n* = 12) [absolute and percent]

Participant characteristics (*n* = 12)		
	Number	[%]
Gender		
Male	11	91.7
Female	1	8.3
Work experience as surgical trainee (months)		
0–12	1	8.3
12–24	1	8.3
24–36	3	25.0
36–48	7	58.3
Previous experience as main surgeon using laparoscopic procedures (number of occasions)		
0–99	6	50.0
100–199	1	8.3
200–299		
300–400	5	41.7
Previous experience as assisting surgeon using laparoscopic procedures (number of occasions)		
0–99	4	33.3
100–199	3	25.0
200–299	2	16.7
300–400	3	25.0
Previous training in technical laparoscopic simulation (hours)		
None		
1–24	5	41.7
25–49	3	25.0
50–74	4	33.3
Overall experience in playing computer games (years)		
None		
10>	6	50.0
10<	6	50.0
Overall experience in fine motoric activities; handcraft, needlework, play of music instruments, or other activities (years).		
None		
10>	10	83.3
10<	2	16.7

% Percentage of

**Fig. 2:**
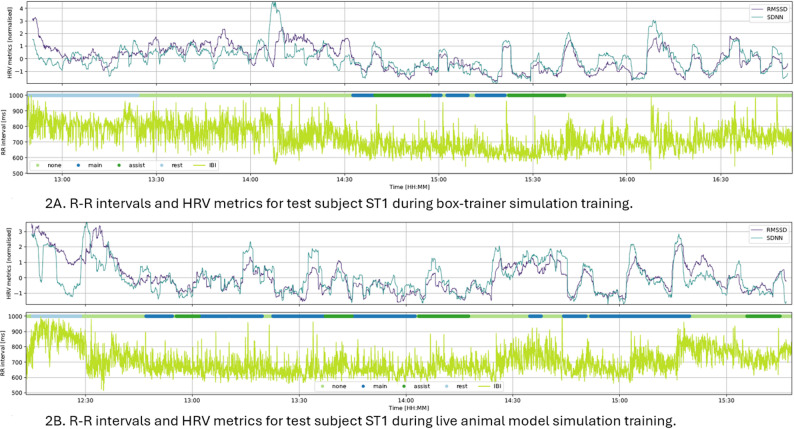
**A** The R-R intervals and HRV metrics for one participant (ST1) during box-trainer simulation training. **B** The R-R intervals and HRV metrics for one participant (ST1) during live animal model simulation training. The SDNN = standard deviation of all R-R intervals in ms, and the RMSSD = square root of the mean of the sum of the squares of differences between adjacent R-R intervals in ms. The R-R interval = the time between two successive R waves in ms. The simulation activities segments are described in different colours, were main = acting as main surgeon, assist = acting as assisting surgeons, rest = resting, and none = non-simulation activity

**Fig. 3:**
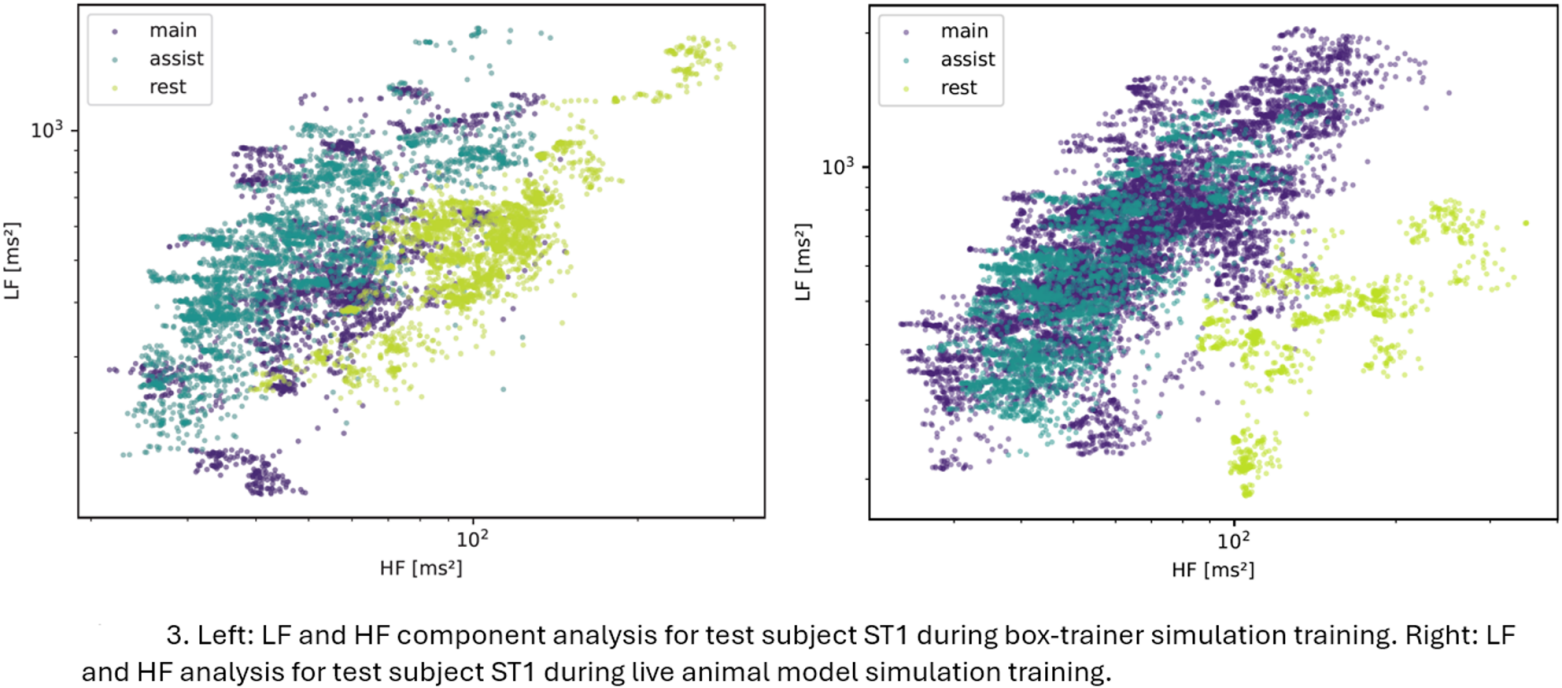
The LF and HF component analysed using the analysis methods by von Rosenberg et al. 2017 [30]. The scatter plot on the left shows the data for one participant (ST1) during box-trainer simulation training, while the scatter plot on the right shows during live animal model simulation training. LF = low-frequency power in ms^2^, HF = high-frequency power in ms^2^. The simulation activities are described in different colours, were main = acting as main surgeon, assist = acting as assisting surgeons and rest = resting

A statistical difference in stress response as measured by the HRV metrics was not found between the simulation-based training in the skills lab compared to the training in operating room settings. However, the HRV metrics SDNN and RMSSD both showed marked changes from resting condition. Fig. 2A and B shows the HRV metrics of one participant (ST1) during box-trainer simulation training, and during the operation on live animal models. The figures illustrate how the SDNN and RMSSD fluctuate during different simulation training activities. The lower part of the Fig. 2A and B illustrates the R-R interval data. Fig. 3 shows a scatter plot of the LF and HF power or amplitude analysed in accordance with the proposed methods by von Rosenberg et al. 2017 [30]. Stress is categorised by characteristic areas in the two-dimensional scatter plot of LF and HF power or amplitude. A high LF combined with a low HF indicates high stress, whereas a low LF and high HF indicate low stress. The scatter plot on the left shows the analysis for one participant (ST1) during box-trainer simulation training, and on the right shows analysis during live animal model simulation training. A qualitative analysis of the scatter plot in Fig. 3 shows that the data points are placed at high LF and at low HF for when the participant ST1 was acting as the main surgeon training on live animal in the operating room, which indicates high stress. In comparison, in resting conditions the data points are placed at low LF and at high HF, indicating low stress.

Salivary cortisol levels were significantly higher during live animal simulation training compared to resting conditions. Mean STAI scores increased during simulation activities in both training environments, showing statistically significant differences from resting conditions. The physiological measures and self-reported stress scores are presented in Table 2.

**Table 2:** Descriptive statistics for all stress response measurements during simulation-based training of advanced procedure training in laparoscopy

Stress measures	Resting conditions before box-trainer simulation	Box-trainer simulation		Resting conditions before live animal model simulation	Live animal model simulation		Number of participants
	Mean ± SD	Mean ± SD	*p*-value	Mean ± SD	Mean ± SD	*p*-value	N
HRV metrics							
SDNN (ms)	66.8 ± 24.6	58.6 ± 13.8		63.2 ± 21.3	48.7 ± 20.9		11#
RMSSD (ms)	39.5 ± 18.7	34.1 ± 13.6		34.2 ± 16.1	21.1 ± 13.1*	*p* = 0.013	11#
Stress hormone activity							
Saliva cortisol (nmol/L)	2.75 ± 2.97	4.51 ± 3.07		3.5 ± 3.08	9.07 ± 7.09*	*p* = 0.036	11#
Self-reported stress scores							
STAI-6(score)	8.3 ± 2.5	11.5 ± 3.1*	*p* = 0.049	8.3 ± 2.4	12.5 ± 3.5*	*p* = 0.013	12

*Mean* arithmetic mean,* SD* Standard deviation, *p* *P-*value, *N* Number of participants, Saliva cortisol in nmol/L, *STAI-6* State-Trait Anxiety Inventory, the 6 item questionnaire, HRV variables: *SDNN* Standard deviation of all R-R intervals in ms, *RMSSD* square root of the mean of the sum of the squares of differences between adjacent R-R intervals in ms

#One participant with missing data is excluded

*Significant compared to resting conditions, *p* < 0.05

### Qualitative findings

The qualitative contents analysis generated several themes on trainees’ stress experiences related to training in both simulation environments. Three dominating themes were identified across both simulation settings: realism and functional task alignment [39], the perceived risk level, and interpersonal dynamics in the simulation environment.

#### Realism

Trainees described how the realism of the training environment impacted on their psychological stress levels. The trainees reported of being more stressed when the simulation tasks felt real. One trainee expressed how the operating room environment impacted his psychological stress levels:

*“I felt stressed because it felt more real (training in the operating room settings)*,* so it affected me psychologically.” (ST3).*

Another trainee expressed the practical benefits of having realistic simulations, which allowed trainees to practice suturing and handling tissue. This approach generated excitement that was more engaging than using box-trainers alone:

*“There’s something about handling tissue*,* pulling and feeling things. I’ve never touched a spleen before; it’s exciting” (ST11).*

Trainees expressed the functional task alignment value of training with the box-trainer:

*“I feel like there’s nothing you can do wrong here (the box-trainer)*,* but you still get to practice those sutures and techniques*,* both sewing and handling tissue” (ST6).*

Several participants described that they were surprised by the anatomical differences between animal organs and human bodies that required trainees to adapt their techniques, which could be stressful but also educational:

*“The surprise is the anatomical difference between the simulator here (a pig) and the human body*,* which means you have to create a new understanding of the anatomical structures in the operating field.” (ST10).*

Another recalled how this would affect the techniques:

*“It was abnormally difficult to place those sutures on the pig. The technical aspect became more difficult than it would have been in a real patient*,* so I stressed with getting it right technically*,* and then I got more stressed overall.” (ST9).*

#### Perceived risk level

Trainees suggested that their stress levels increased significantly when the stakes were perceived as high, such as working on a live animal model. Trainees described how the realism of working with a live pig contributed to a sense of seriousness and stress during the simulation training. This heightened stress was reported to increase focus and sharpen the performance:

“*My stress level increases when the stakes are high. I feel more focused*,* because when thinking of everything that could go wrong makes the stress level go up. But you’re generally quite focused regardless*,* but extra in this situation (operating live animals).” (ST1).*

In contrast to working on a live animal model, trainees described how they had lower stress experiences with the box-trainers, where the consequences of mistakes were not as severe:

*“The stress was very low on the box-trainer because it’s a very simulated setting. I felt very little stress. The stakes are very low if you do something very wrong. That’s where my stress comes from in an operative situation. On the live pig*,* the stress was still relatively low but*,* significantly higher (than the box-trainer) because you get the real feedback from the bleeding you caused.” (ST10).*

Trainees’ stress levels were reported to be influenced by the degree of control over the operative situation:

*“The pig yesterday wasn’t in great shape*,* but I still felt we had control of the situation. It’s clear that you get a stressed when the abdomen fills up with a lot of blood. It was a very uncomfortable situation.” (ST6).*

On the other end, the trainees expressed how they did not feel stressed when training on the box-trainer:

*“No*,* I didn’t get stressed! That’s the problem with the simulator; it’s not like the patient dies if you don’t seal the splenic artery well enough.” (ST10).*

#### Interpersonal dynamics in simulation training environment

The impact of interpersonal dynamics in the simulation environments was reported to increase the stress levels, leading trainees to be concerned about making mistakes that could reduce the practice time available for their colleagues:

*“What I found most stressful was that I didn’t want to take up other people’s time*,* that I shouldn’t mess up so they would have less time to practice themselves.” (ST4).*

The presence and actions of instructors and peers was reported to influence the trainees’ stress levels. Trainees recalled how the supportive and structured guidance reduced stress and improved the learning experience:

*“Yes! I think you might need to be under a bit pressure to develop yourself as surgeon… Not too much pressure*,* but a little! It’s very reassuring if someone (instructors or peers) stands next to you and guides you on what to do.” (ST6).*

## Discussion

### Physiological stress measures and perceived stress

Physiological and self-reported stress measures revealed no significant differences between simulation modalities or environments. However, qualitative interview data indicated that trainees experienced higher perceived stress during procedures involving live animal models in the operating room environment. This divergence may reflect the multifaceted nature of stress responses. Previous research show that physiological stress response as measured by HRV metrics and cortisol levels, can occur rapidly and automatically [16, 40], whereas perceived stress is influenced by cognitive appraisal, previous experiences, coping mechanism and other psychological elements, and may develop more gradually [13, 15, 41]. In a simulation training setting, both types of stress processes can occur simultaneously and yet manifest differently, potentially leading to inconsistent outcomes [10, 42].

A significant reduction in RMSSD and increase in saliva cortisol levels were observed when comparing rest to simulation activities in the operating room environment. Although participants were experienced surgical trainees, they were performing unfamiliar procedures in a novel setting, likely triggering autonomic nervous system activation. This response may reflect their appraisal of the situation as either a challenge or a threat, potentially explaining the observed physiological changes [15, 43]. Similar patterns of autonomic activation have been reported in previous simulation studies, where transitions from rest to active simulation activities were associated with marked changes in HRV metrics [44, 45] and in cortisol levels [3].

### Realism, functional task alignment and perceived risk in simulation training

Participants reported elevated stress when transitioning from rest to simulations in a realistic operating room setting. The use of live tissue, authentic surgical equipment, and attire likely contributed to this response. This aligns with prior research showing that high-fidelity simulations, featuring realistic sounds, equipment, social dynamics, and audience presence, can elicit significant physiological and psychological stress [46, 47]. The simulation environment provided trainees with an immersive experience closely resembling real surgical settings, including stress-inducing factors commonly encountered in clinical practice [48, 49].

Trainees identified simulation realism and functional task alignment as key factors influencing stress responses. Box-trainer simulations, perceived as less realistic and lower in task alignment, elicited less stress. Box-trainers are designed primarily for technical skill development, such as repetition, error correction, and precision, not necessarily intended to replicate clinical realism [4]. These findings align with existing research on benchtop model simulators [5, 45]. In contrast, simulations involving live animal models were viewed as highly realistic, with task demands closely aligned to clinical procedures, and were associated with higher stress response levels. These perceptions are align with existing literature, which highlights how live animal models can effectively replicate the emotional, cognitive, and procedural challenges encountered in actual surgical practice [50].

Trainees consistently identified the risk of uncontrolled bleeding during live animal model simulations as a major stressor. Elevated stress was perceived to impair their ability to manage intraoperative complications. These simulations expose trainees to acute challenges such as bleeding management [50], aligning with prior research showing strong stress responses to bleeding among clinicians [51], and suggesting autonomic activation during these simulations [52, 53].

Although simulations involving animal models and realistic environments elicited elevated stress levels, trainees reported improved focus on their performance. In the present study, simulation realism appears to enhance participants’ performance, consistent with findings from high-fidelity simulation research [3, 46, 54]. These results suggest that increased stress, induced by realistic simulation environment, may enhance focus and engagement, particularly beneficial in surgical training [55].

### Interpersonal dynamics in simulation training environment

Trainees reported elevated stress linked to interpersonal dynamics within the simulation environment. While realistic simulation environments are effective for evaluation of performance under pressure and assessing surgical skill competency, they may also expose deficiencies in both technical and non-technical skills [56, 57]. This exposure may place trainees in a vulnerable position by revealing their actual level of surgical competence. The close involvement of instructors and peers during operating room simulations, compared to skills lab training, may contribute to heightened stress perceptions [9, 58].

Instructor supervision was perceived to contribute to both eustress and distress. While the presence of instructors may elevate stress and negatively affect performance, it can also enhance psychological safety and support learning through expert guidance [53, 58, 59]. These benefits of having instructors in the simulation environment have been demonstrated to enhance educational and learning outcomes [60, 61].

### Implications

The findings of this study may guide the design of simulation-based surgical training by emphasising the need to calibrate environments so that stress levels align with trainees’ experience and learning objectives. Low-stress scenarios, such as box-trainers, support early skill acquisition, while high-stress scenarios, such as live animal models or realistic operating room environments, can support decision-making under pressure, contextual learning, and resilience. Measuring stress responses is the first step, enabling targeted feedback and task adjustments to keep stress within productive ranges. Structured reflection sessions are the next step, allowing trainees to assess whether stress was detrimental or motivational and thereby strengthening awareness of stress management strategies and the development of coping skills.

Furthermore, instructors can develop strategies to support trainees exhibiting excessive stress during task performances. These individuals should be given extra attention and followed up as needed throughout the course, with feedback communicated to their institution to ensure appropriate support measures are implemented.

Finally, future research should explore how new technology such as AI-driven virtual reality and mixed reality platforms can serve as viable alternatives to live animal models [62, 63]. These technologies can replicate the realism of clinical environments while avoiding ethical and logistical challenges. Moreover, they can be tailored to simulate specific stressors and learning scenarios, supporting individualised and scalable training. Integrating high-fidelity simulations with digital platforms has the potential to reduce reliance on ethically sensitive models while preserving educational effectiveness.

### Limitations

With the limitation of a small sample size, a statistical difference in the HRV metrics may have been difficult to observe. However, a significant difference in RMSSD during the simulation training environment compared to resting conditions was observed, suggesting sufficient statistical power in the data set. Additional analyses of the HRV data were conducted using various analytical approaches, which confirmed the study results.

## Conclusions

In this study, no differences in physiological stress responses were observed between simulation training on the box-trainer and procedural training on live animal models. Interviews, however, indicated that trainees experienced greater stress during advanced procedural training in realistic operating room environments compared to skills laboratories. As a result of heightened stress, trainees focused more on their performance during simulation sessions. These findings suggest that realistic simulation environments, although more stressful, enhance the training process for experienced trainees and should be incorporated into surgical training programs.

## Supplementary Information

Additional file 1. Participant background questionnaire.

Additional file 2. Table S1. Supplementary Table 1. Description of the simulations in accordance with Cheng et al. 2016. Key Elements to Report for Simulation-Based Research.

Additional file 3. Reporting in accordance with the STROBE with simulation-based research extension.

Additional file 4. Table S2. Reporting in accordance with the COREQ guidelines-checklist.

Additional file 5. STAI-6 questionnaire form.

Additional file 6. The interview guide.

## Data Availability

All data relevant to the study are included in the article or uploaded as supplemental information. Key findings of the research are included in the study. Both interview transcripts and unprocessed ECG data are available from the main author Maria Suong Tjønnås at email: [lethi@ntnu.no](mailto: lethi@ntnu.no) upon reasonable request. The interview data is in Norwegian and ECG data is in EDF file format.

## Abbreviations

AI: Artificial intelligence
ANOVA: Analysis of variance
ANS: Autonomic nervous system
ECG: Electrocardiogram
HPA: Hypothalamic-pituitary-adrenal
HRV: Heart rate variability
Hz: Hertz
HF: High frequency
LF: Low frequency
ms: Milliseconds
REC: Regional committees for medical and health research ethics
RMSSD: Root mean square of successive differences of R-R intervals
R-R interval: The time elapsed between two successive R waves of the QRS signal on the electrocardiogram
R-waves: Representing ventricular depolarisation
SDNN: Standard deviation of the R-R intervals
STAI-6: State Trait Anxiety Inventory-6 short version
STROBE: Strengthening the reporting of observational studies in epidemiology
VR: Virtual reality
