# Supplementary material for: Stress responses and experiences of surgical trainees in simulation-based training of advanced laparoscopic procedures in highly realistic environments
Source: Adv Simul (Lond). 2026 Jan 9;11:6. doi: 10.1186/s41077-025-00400-z (PMC12882263; doi:10.1186/s41077-025-00400-z)
Supplement: Supplementary file 1 — Additional file 1. Participant background questionnaire. [file 41077_2025_400_MOESM1_ESM.docx]

Questionnaire for the research project

STRESS RESPONSES IN SIMULATION-BASED TRAINING OF ADVANCED LAPAROSCOPIC SURGICAL PROCEDURES

**Introduction**

**Initials: __________________________**

**Age: ____________**

**Sex: ____________**

**Height and weight (for the calculation of physiological parameters): _________________**

**Date: ______________________________**

1. **Background**

1a. Describe your professional practice postgraduate?

______________________________________________________________________________________________________________________________________________________________________________________________________________________________________________________

1b. What is your current medical discipline?

__________________________________________________________________________________

1c. Do you have additional medical disciplines?

1. **Experience with simulation-based training**

2a. What type of simulation-based training have you done in the past?

____________________________________________________________________________________________________________________________________________________________________

2b. In what context ?

__________________________________________________________________________________

2c. For how long did the training last (hours, days, weeks)?

1. **Experience with other form of digital training**

3a. Do you have any experience with other forms of digital training (e.g. online courses, apps, etc.)?

____________________________________________________________________________________________________________________________________________________________________

3b. In what context?

­__________________________________________________________________________________

3c. For how long did the training last for (hours, days, weeks)?

__________________________________________________________________________________

1. **Experience with laparoscopic surgery and techniques**

4a. Have you previously trained on laparoscopic box-trainers or other simulators? If yes, for how long did the training last for (hours, days, weeks)?

__________________________________________________________________________________

**5. Experience with laparoscopic surgery and techniques**

5a. How many laparoscopic operations have you observed (approximate number)?

__________________________________________________________________________________

5b. How many laparoscopic operations have you assisted (approximate number)?

__________________________________________________________________________________

5c. How many laparoscopic operations have you performed as main operator (approximate number)?

**6. Experience with computer games**

Do you play or have you played computer games (now/in previous years)?

5a. Describe the amount of time you have spent playing computer games (approx. number of years/hours per week)?

**7. Experience with e.g., handwork, knitting, needlecrafts, playing string instruments or other activities that requires a high degree of motoric precision?**

7a. Describe the amount of time you have spent (approx. number of years/hours per week)?

__________________________________________________________________________________

**8. General health assessment**

8a. How would you describe your general health (give an overall brief assessment)?

____________________________________________________________________________________________________________________________________________________________________8b. How would you describe your present state of stress level ( i. e., none, restless, nervous, anxious, problems with sleep quality, etc.,)?

____________________________________________________________________________________________________________________________________________________________________8c. Do you know of any physiological abnormalities that could influence the neurological or cardiological outcomes measures (e. g., hereditary irregular heart rhythms, hormonal disorders, etc.)?

__________________________________________________________________________________

8d. Do you have a known imbalance in your cortisol level?

________________________________________________________________________________

8e. Do you have motoric limitations/other limitations in fine motor skills, eyesight or hand-eye coordination?

__________________________________________________________________________________

**9. Physical activity (related to heart rate variability measurements)**

9a. How would you describe your physical fitness? (give an overall brief assessment)

____________________________________________________________________________________________________________________________________________________________________9b. Have you previously been involved in organized sports training? If so, what type of sport?

__________________________________________________________________________________

9c. Do you exercise on regular base ? If so, what type of exercise?

9d. If so, how frequently do you exercise?

- less than once a month
- 1-2 times a month
- 1-2 times a week
- 3-4 times a week
- more than 4 times a week

9e. What is your resting heart rate (approx.)?
