# Supplementary material for: Stress responses and experiences of surgical trainees in simulation-based training of advanced laparoscopic procedures in highly realistic environments
Source: Adv Simul (Lond). 2026 Jan 9;11:6. doi: 10.1186/s41077-025-00400-z (PMC12882263; doi:10.1186/s41077-025-00400-z)
Supplement: Supplementary file 2 — Additional file 2. Table S1. Supplementary Table 1. Description of the simulations in accordance with Cheng et al. 2016. Key Elements to Report for Simulation-Based Research. [file 41077_2025_400_MOESM2_ESM.docx]

Supplementary Table 1. Description of the simulator type, orientation to simulators and environment, simulation-based training, and feedback/debriefing. Cheng, A., Kessler, D., Mackinnon, R. *et al.* Reporting guidelines for health care simulation research: extensions to the CONSORT and STROBE statements. *Adv Simul* 1, 25 (2016).

| **Simulator type** | | |
| --- | --- | --- |
| Simulator make and model* | Box-trainer (Pulsatile organ perfusion (P.O.P) box-trainer, Optimist, Innsbruck, Austria). | Training operations using animal models as human patient simulators. |
| Simulator functionality* | The P.O.P -trainer, a box-trainer which provided training on artificial organ models and real tissue using authentic laparoscopic instruments. The simulator had a top lid with entry ports for trocars. Both artificial organ models and porcine tissue (stomach and small intestines) were used to facilitate the simulation tasks. Limitations: the pulsatile function which simulates circulation of liquids in real tissue was not employed. | An anesthetized pig was used as a human patient simulator. It was anesthetized during the whole operation. The training adhered to the ethical guidelines for the use of animals in research as described in the Directive 2010/63/EU of the European Parliament and of the Council of 22. September 2010 on the protection of animals used for scientific purposes. |
| **Orientation to simulators and environment** | | |
| Participants orientation to the simulator ahead of the simulation sessions* | Trainees were given outline of the laparoscopic procedures, and practical guide in using the surgical instruments, and instructions on how to work in pairs. | Trainees were given outline of the laparoscopic procedures, and practical guide in using the surgical instruments, and operating room facilities, and instructions on how to work teams. |
| Participant orientation to the environment ahead of the simulations session* | Trainees were assigned to one of five simulators, which were placed on bench tops and connected to a laparoscopic tower. Instructors were available on site, and trainees were allowed to engage in conversations or observations during training. | The trainees were divided into operating teams of three or four persons, where one acted as the main surgeon, another as the main assistant surgeon, and the remaining as assistant surgeons providing technical support for the main surgeon. A surgeon instructor guided the trainees through the surgical procedures. |
| Group vs. individual practice* | The trainees performed the simulation tasks in pairs. | The trainees performed the operation in group of three or four. |
| **The simulation-based procedures training** | | |
| The simulation-training procedures | Simulation training:   1. Exercises to familiarise the trainee with specific procedural suture and cutting techniques conducted on pig stomachs and small intestines. 2. Conducting anastomosis and resection procedures using pig tissue (stomachs and small intestines). | Live operation training:   1. Pre-operation preparation procedures. 2. Placement of trocars in preparation for the operation. 3. Conducting anastomosis and/or resection procedures on the pig stomach and small intestines. |
| Learning objectives* | (1) Get familiarised with the laparoscopic instruments, and practise specific procedural suture and cutting techniques on pig stomach and small intestines.  (2) Conducting suture and cutting techniques.  (3) Conducting an anastomosis or a resection exercise on pig stomach on small intestines. | (1) Get familiarised with the laparoscopic instruments and other instruments used in the procedures  (2) Conducting an anastomosis or a resection procedure on pig stomach on small intestines.  (3) Control of complications during the procedures such as bleedings or perforations of tissue walls. |
| Requirements/assessment* | Accomplishing an end-to-end anastomosis exercise using laparoscopic techniques. | 1. Accomplishing an end-to-end anastomosis procedure using laparoscopic techniques. 2. Management of intraoperative complications in connection of the |
| Frequency/repetitions* | No restrictions on number of repetition, but time restrictions applied. | Time restrictions of 1h applied. |
| **Feedback/debriefing** | | |
| Feedback* | Instructors gave feedback on technical performance and provided guidance on techniques. | Instructors gave feedback on the procedural performance and provided guidance in managing perioperative complications, new techniques or the use of unfamiliar surgical instruments. |
| Instructor presence* | Two to three surgical instructors were present throughout all simulation sessions. | One surgical instructor was present for each surgical team throughout the operation session. |
| Instructor characteristics* | Instructors were medical educators with more than 15 years of experience. Instructors were general surgeons with specialisation in gastroenterology and gynaecology, with more than 25 years of laparoscopic experience. | Instructors were medical educators with more than 15 years of experience. Instructors were general surgeons with specialisation in gastroenterology, with more than 25 years of laparoscopic experience. |
| Non-simulation interventions and adjuncts* | Lectures on laparoscopic topics, laparoscopic techniques, advanced laparoscopic procedures, and the learning outcomes using box-trainers and in performing operations were given prior to the simulation-training sessions and operations sessions. | |

*Elements from Cheng et al. (2016). Table 3 Key Elements to Report for Simulation-Based Research.
