# Supplementary material for: Stress responses and experiences of surgical trainees in simulation-based training of advanced laparoscopic procedures in highly realistic environments
Source: Adv Simul (Lond). 2026 Jan 9;11:6. doi: 10.1186/s41077-025-00400-z (PMC12882263; doi:10.1186/s41077-025-00400-z)
Supplement: Supplementary file 3 — Additional file 3. Reporting in accordance with the STROBE with simulation-based research extension. [file 41077_2025_400_MOESM3_ESM.docx]

**STROBE extension for simulation-based research (SBR)**

Reference: Cheng A, Kessler D, Mackinnon R, Chang TP, Nadkarni VM, Hunt EA, Duval-Arnould J, Lin Y, Cook DA, Pusic M, Hui J, Moher D, Egger M, Auerbach M. Reporting guidelines for health care simulation research: extensions to the CONSORT and STROBE statements. Adv Simul. 2016 Jul 25;1:25.

| **Item number** | **STROBE description (Observational studies)** | **Extension for SBR** |
| --- | --- | --- |
| Title and abstract | a. Indicate the study’s design with a commonly used term in the title or the abstract. | In abstract or key terms, the MESH or searchable keyword term must have the word simulation or simulated. |
|  | b. Provide in the abstract an informative and balanced summary of what was done and what was found. |  |
| **Introduction** | | |
| Background/rationale | Explain the scientific background and rationale for the investigation being reported. | Clarify whether simulation is subject of research or investigational method for research. |
| Objectives | State specific objectives, including any prespecified hypotheses. |  |
| **Methods** | | |
| Study design | Present key elements of study design early in the paper. |  |
| Setting | Describe the setting, locations, and relevant dates, including periods of recruitment, exposure, follow-up, and data collection. |  |
| Participants | a. Cohort study: give the eligibility criteria and the sources and methods of selection of participants. Describe methods of follow-up. |  |
|  | Case–control study: give the eligibility criteria and the sources and methods of case ascertainment and control selection. Give the rationale for the choice of cases and controls. |  |
|  | Cross-sectional study: give the eligibility criteria and the sources and methods of selection of participants. |  |
|  | b. Cohort study: for matched studies, give matching criteria and number of exposed and unexposed. |  |
|  | Case–control study: for matched studies, give matching criteria and the number of controls per case. |  |
| Variables | Clearly define all outcomes, exposures, predictors, potential confounders, and effect modifiers. | Describe the theoretical and/or conceptual rationale for the design of the intervention/exposure. |
|  | Give diagnostic criteria, if applicable. | Describe the intervention/exposure with sufficient detail to permit replication. |
|  |  | Clearly describe all simulation-specific exposures, potential confounders, and effect modifiers. |
| Data sources/measurement | For each variable of interest, give sources of data and details of methods of assessment (measurement). | In describing the details of methods of assessment, include (when applicable) the setting, instrument, simulator type, timing in relation to the intervention, along with any methods used to enhance the quality of measurements. |
|  | Describe comparability of assessment methods if there is >1 group. | Provide evidence to support the validity and reliability of assessment tools in this context (if available). |
| Bias | Describe any efforts to address potential sources of bias. |  |
| Study size | Explain how the study size arrived. |  |
| Quantitative variables | Explain how quantitative variables were handled in the analyses. If applicable, describe which groupings were chosen and why. |  |
| Statistical methods | a. Describe all statistical methods, including those used to control confounding. | Clearly indicate the unit of analysis (e.g., individual, team, system), identify repeated measures on subjects, and describe how these issues were addressed. |
|  | b. Describe any methods used to examine subgroups and interactions. |  |
|  | c. Explain how missing data was addressed. |  |
|  | d. Cohort study: if applicable, explain how loss to follow-up was addressed. |  |
|  | Case–control study: if applicable, explain how matching of cases and controls was addressed. |  |
|  | Cross-sectional study: if applicable, describe analytical methods taking account of sampling strategy. |  |
|  | e. Describe any sensitivity analyses. |  |
| **Results** | | |
| Participants | a. Report the numbers of individuals at each stage of the study (e.g., numbers potentially eligible, examined for eligibility, confirmed eligible, included in the study, completing follow-up, and analysed). |  |
|  | b. Give reasons for nonparticipation at each stage. |  |
|  | c. Consider use of a flow diagram. |  |
| Descriptive data | a. Give characteristics of study participants (e.g., demographic, clinical, social) and information on exposures and potential confounders. | In describing characteristics of study participants, include their previous experience with simulation and other relevant features as related to the intervention(s). |
|  | b. Indicate the number of participants with missing data for each variable of interest. |  |
|  | c. Cohort study: summarize follow-up time (e.g., average and total amount). |  |
| Outcome data | Cohort study: report numbers of outcome events or summary measures over time. |  |
|  | Case–control study: report numbers in each exposure category or summary measures of exposure. |  |
|  | Cross-sectional study: report numbers of outcome events or summary measures. |  |
| Main results | a. Give unadjusted estimates and, if applicable, confounder-adjusted estimates and their precision (e.g., 95 % confidence intervals). | For assessments involving >1 rater, interrater reliability should be reported. |
|  | Make clear which confounders were adjusted for and why they were included. |  |
|  | b. Report category boundaries when continuous variables were categorized. |  |
|  | c. If relevant, consider translating estimates of relative risk into absolute risk for a meaningful time period. |  |
| Other analyses | Report other analyses done (e.g., analyses of subgroups and interactions and sensitivity analyses). |  |
| **Discussion** | | |
| Key results | Summarize key results with reference to study objectives. |  |
| Limitations | Discuss limitations of the study, taking into account sources of potential bias or imprecision. Discuss both direction and magnitude of any potential bias. | Specifically discuss the limitations of SBR. |
| Interpretation | Give a cautious overall interpretation of results considering objectives, limitations, multiplicity of analyses, results from similar studies, and other relevant evidence. |  |
| Generalizability | Discuss the generalizability (external validity) of the study results. | Describe generalizability of simulation-based outcomes to patient-based outcomes (if applicable). |
| **Other information** | | |
| Funding | Give the source of funding and the role of the funders for the present study and, if applicable, for the original study on which the present article is based. | List simulator brand and if conflict of interest for intel |
