# Supplementary material for: Stress responses and experiences of surgical trainees in simulation-based training of advanced laparoscopic procedures in highly realistic environments
Source: Adv Simul (Lond). 2026 Jan 9;11:6. doi: 10.1186/s41077-025-00400-z (PMC12882263; doi:10.1186/s41077-025-00400-z)
Supplement: Supplementary file 4 — Additional file 4. Table S2. Reporting in accordance with the COREQ guidelines-checklist. [file 41077_2025_400_MOESM4_ESM.docx]

**Table S1. Report in accordance with the COREQ guidelines-checklist for reporting qualitative research** (1)**.**

| No item | Description |
| --- | --- |
| ***Domain 1: Research team and reflexivity*** |  |
| **Personal characteristics** |  |
| 1. Interviewer/facilitator | MST conducted all interviews. |
| 2. Credentials | The first author and scientific researcher MST, was a PhD student, holds a MSc. in Physiology, is a registered nurse (RN), with research experience of more than 15 years; SOO holds a PhD and is a senior researcher with more than 15 years of research experience in health care services and socioeconomics. MS holds a PhD in Physiology and is a senior researcher with more than 30 years of research experience in physiology. JT holds a PhD in Mathematic and Cybernetics and work as researcher with more than 15 years of research experience. SM holds a PhD in Medical Technology and work as researcher with more than 15 years of research experience. CV holds a PhD in Medical Technology and work as researcher with more than 15 years of research experience. GJ is a senior surgeon consultant and was head surgeon at the university hospital, with more than 30 years of surgical experience, and more than 15 years of experience as surgical training instructor. |
| 3. Occupation | MST is a scientific researcher at SINTEF and PhD student at the Norwegian University of Science and Technology in Trondheim, Norway. |
| 4. Gender | MST, SOO, CV and MS are female. JT, SM and GJ are male. |
| 5. Experience and training | MST had experience in research from different areas including quantitative and qualitative research, with more than 15 years within the physiological research field, and more than 5 years of clinical experience. |
| **Relationship with participants** |  |
| 6. Relationship established | There was no relationship between researcher and the participants. The researcher and the participants had never met prior to the courses, and there were no dependence issues between researcher and participants. |
| 7. Participant knowledge of the interviewer | Participants were given information about the researcher and study goals through written information about the project ahead of the courses. The participants who asked for more information, were given additional information. |
| 8. Interviewer characteristics | The article includes information about the professional background of the interviewer. The main interest of MST in the topic was grounded in her PhD studies on stress and laparoscopic simulation-based training in surgical trainees. |
| ***Domain 2: study design*** |  |
| **Theoretical framework** |  |
| 9. Methodological orientation and theory | The framework of the study were cognitive stress theories, biological stress response theories and surgical simulation-based training theories. The qualitative analysis method used was qualitative content analysis topics from Graneheim and Lundman (2004)(2). |
| **Participant selection** |  |
| 10. Sampling | Purposive sampling. We aimed for a group of advanced surgical trainees which represented a realistic sample regarding previous laparoscopic simulation experiences. All participants were enrolled in surgical training courses in advanced laparoscopic procedures. No participants withdrew from the study. |
| 11. Method of approach | The participants were approached by email and face-to-face by the first author. An oral invitation was given at the beginning of each course by the course instructors. Participants received written information about the study and had the opportunity to ask questions ahead of the interviews and data collection. |
| 12. Sample size | 12 participants were included in the final analysis. |
| 13. Non-participation | None of the included participants withdrew. |
| **Setting** |  |
| 14. Setting of data collection | The interview data were collected in a private office or a sitting group at the training facilities. |
| 15. Presence of non-participants | No one else was present besides the participants and the researcher MST during interviews. |
| 16. Description of sample | The sample consisted of advanced surgical trainees with more than 2 years of specialist training, and surgeon instructors with more than 30 years of experience within surgery. |
| **Data collection** |  |
| 17. Interview guide | The interview guide is provided as additional file 6. The guide was piloted and published in Tjønnås et al. 2022. This study used an adapted version of this interview guide. |
| 18. Repeat interviews | No repeated interviews were carried out. |
| 19. Audio/visual recording | All interviews were audio recorded and stored in accordance with recommendations of the regional ethics committee. |
| 20. Field notes | N/A |
| 21. Duration | The interviews lasted for 20-60 minutes. |
| 22. Data saturation | Data saturation was reached when no new themes were identified through preliminary analysis. |
| 23. Transcripts returned | Transcripts were not returned to participants; however, the interviews were summarized, and participant could orally comment and correct their statements. |
| ***Domain 3: analysis and findings*** |  |
| **Data analysis** |  |
| 24. Number of data coders | The main author coded the data in collaboration with a co-author |
| 25. Description of the coding tree | Themes were derived from data. No coding tree was used. |
| 26. Derivation of themes | Themes were derived from the data. |
| 27. Software | To manage data the method described by Ose (2016) was used (3). |
| 28. Participant checking | The participants did not provide feedback on the overall findings. However, at the end of the interviews, a summary of interview was given, and participants could comment and correct the interview contents. |
| **Reporting** |  |
| 29. Quotations presented | Themes are presented and illustrative quotations /excerpts are presented in the results section. The excerpts are identified by participant number (surgical trainee = ST, numbers: 1, 2, 3…n) |
| 30. Data and findings consistent | The presented data and findings are consistent. These are presented in the results section in the main manuscript text. |
| 31. Clarity of major themes | Major themes are presented in the results section. |
| 32. Clarity of minor themes | Minor themes are presented in the results section. |

1. Tong A, Sainsbury P, Craig J. Consolidated criteria for reporting qualitative research (COREQ): a 32-item checklist for interviews and focus groups. International Journal for Quality in Health Care. 2007;19(6):349–57.

2. Graneheim UH, Lundman B. Qualitative content analysis in nursing research: concepts, procedures and measures to achieve trustworthiness. Nurse Educ Today. 2004 Feb;24(2):105–12.

3. Ose SO. Using Excel and Word to Structure Qualitative Data. Journal of Applied Social Science. 2016;10(2):147–62.
