# Supplementary material for: Stress responses and experiences of surgical trainees in simulation-based training of advanced laparoscopic procedures in highly realistic environments
Source: Adv Simul (Lond). 2026 Jan 9;11:6. doi: 10.1186/s41077-025-00400-z (PMC12882263; doi:10.1186/s41077-025-00400-z)
Supplement: Supplementary file 5 — Additional file 5. STAI-6 questionnaire form. [file 41077_2025_400_MOESM5_ESM.docx]

**Additional file 5:**

**The State-Trait Anxiety Inventory (STAI)**

**Modified version of Marteau, T.M. and Bekker, H. (1992), The development of a six-item short-form of the state scale of the Spielberger State—Trait Anxiety Inventory (STAI). British Journal of Clinical Psychology, 31: 301-306.**

Read each statement and then circle the most appropriate number to the right of the statement to indicate how you feel right *now*, at this moment. There are no right or wrong answers. Do not spend too much time on any one statement but give the answer which seems to describe your present feelings best.

|  | Not at all | Somewhat | Moderately | Very much |
| --- | --- | --- | --- | --- |
| I feel calm | 1 | 2 | 3 | 4 |
| I am tense | 1 | 2 | 3 | 4 |
| I feel upset | 1 | 2 | 3 | 4 |
| I am relaxed | 1 | 2 | 3 | 4 |
| I feel content | 1 | 2 | 3 | 4 |
| I am worried | 1 | 2 | 3 | 4 |

Please make sure that you have answered all the questions.
