# Supplementary material for: Stress responses and experiences of surgical trainees in simulation-based training of advanced laparoscopic procedures in highly realistic environments
Source: Adv Simul (Lond). 2026 Jan 9;11:6. doi: 10.1186/s41077-025-00400-z (PMC12882263; doi:10.1186/s41077-025-00400-z)
Supplement: Supplementary file 6 — Additional file 6. The interview guide. [file 41077_2025_400_MOESM6_ESM.docx]

**Interview guide for the study:**

**Stress responses and experiences in simulation-based training course in advanced laparoscopic procedures**

**Introduction:**

**Background and previous experience**

We want to know if you experienced any stress during the three days at this simulation-based training course in advanced laparoscopic procedures, and we want to understand what factors and features of the course you perceived as stressful?

1. We would like to know about your background and your experiences regarding simulation, laparoscopy technique and practice.

*Probes:*

- *Date of birth*
- *Basic medical education, professional practice postgraduate, surgical discipline(s)*
- *Experience with simulation-based training (context, type, length of time and estimated hours spent, simulator modalities)*
- *Experience with laparoscopic surgery (**estimated number of laparoscopic operations observed, assisted, or performed)*
- *Experience with laparoscopic technique and skills (estimated number of hours spent training on simulators, type of laparoscopic simulators, robotic laparoscopic systems)*

1. We would like to know how you divide the time between work in clinical settings and the time you spend learning/training/performing laparoscopy techniques and skills?

*Probes:*

- *Estimated number of hours practicing laparoscopic technique and skill during a regular work week, month, or year.*

1. We would like to know if you have previous experience with computer games.

*Probes:*

- *Familiarity with computer-based gaming*
- *Familiarity with computer-based virtual reality interface*
- *Familiarity with computer-based gaming techniques*
- *Approximately amount of time spent as a player of computer games*
- *Estimated number of years*
- *Estimated hours per week*

1. We would like to know your experience with e.g., handwork, knitting, needlecrafts, playing string instruments or other activities that requires a high degree of motoric precision?

*Probes:*

- *Approximately year(s) of experience?*
- *Hours per week?*

**Main topic:**

**Stress experiences in this simulation-based training course in advanced laparoscopic procedures (trainees overall experience)**

Please, describe your experiences of stress or stressors during this simulation-based training course in advanced laparoscopic procedures.

*Probes:*

*- The course set-up*

- *Stress related to technical demands*
- *Stress related to cognitive demands*
- *Stress related to time pressures*
- *Combination of technique, cognitive demands, and time pressure*
- *Pressures from instructors*
- *Pressures from course participants (training partners)*
- *Self-imposed pressure (personality trait, high /low stakes task, embodied surgeon role, concerns about transferability to real context)*
- *Influences from other participants or instructors*

1. Please, describe what external factors elicited your experience of stress during the SBT course in advanced laparoscopic procedures?

*Probes:*

- *Noise*
- *Parallel conversations*
- *Ambient temperature*
- *Lighting conditions*
- *Your working postures*
- *Psychomotor demands*
- *Task requirements related*
- *Equipment related*
- *Other operation room personnel*

1. Please, describe what internal factors you believe influenced your stress experience during the course?

*Probes:*

- *Personal ambitions*
- *Personality traits*
- *Learning habits*
- *Environmental-specific*
- *Situational-specific*
- *The collaborative relationships with your training partner*
- *Fear of making mistakes or errors*

1. How would you describe the influence your stress experience had on you during this simulation-based training course?

**Stress experiences related to simulation tasks or simulation scenarios/settings**

You have now been training laparoscopic procedures with the simulator modality the P.O.P-trainer and through live operation on an animal model.

1. Please, describe if you experienced any stress while training the laparoscopic procedures?

*Probes: why did/did you not experience stress?*

1. Please, describe what stress experiences you had when training with the P.O.P-trainer and during the operation?
2. Why do you think you experienced stress when training using the P.O.P-trainer or during the operation?

*Probes:*

- *The simulation tasks for the P.O.P- trainer and operation on animal model*
- *Stress related to the computer interface, software, other software related issues*
- *Stress related to the laparoscopic instruments and other equipment issues*
- *Stress related to technical demands* *of simulation tasks*
- *Stress related to cognitive demands* *of simulation tasks*
- *Stress related to time pressures* *of simulation tasks*
- *Combination of technique, cognitive demands, and time pressure*
- *Pressures from instructors*
- *Pressures from course participants (training partners)*
- *Internal pressures/subjective pressures*
- *Influences from other participants or instructors*

1. How would you describe how the stress experience influenced your training or performance of the advanced laparoscopic procedures?

**Closure:**

Do you have any thoughts, perspectives, or related topics you would like to embellish or specify?

**Closing the interview:**

Make a recap of dominant interview themes, ask interviewee to verify or clarify their statements and views.
